# Mindfulness Dampens Cardiac Responses to Motion Scenes of Violence

**DOI:** 10.1007/s12671-017-0799-6

**Published:** 2017-08-31

**Authors:** Artur Brzozowski, Steven M. Gillespie, Louise Dixon, Ian J. Mitchell

**Affiliations:** 1 School of Psychology, University of Birmingham, Birmingham, B15 2TT UK; 2 School of Psychology, Newcastle University, Newcastle, NE1 7RU UK; 3 School of Psychology, Victoria University of Wellington, Po Box 600, Wellington, 6140 New Zealand

**Keywords:** Trait mindfulness, Heart rate, Attention, Emotion, Video

## Abstract

Mindfulness is linked with improved regulatory processes of attention and emotion. The potential benefits of mindfulness are vast, including more positive emotional states and diminished arousal in response to emotional stimuli. This study aims to expand of the current knowledge of the mechanisms of mindfulness by relating the latter to cardiovascular processes. The paper describes two studies which investigated the relationship of trait mindfulness to self-report measures of emotions elicited during a violent video clip and cardiovascular responses to the clip. Both studies recruited male and female participants, mainly university undergraduate students. The clip was 5-min-long and evoked mainly feelings of tension and disgust. In study 1, we found that higher scores for trait mindfulness were associated with increased scores for valence (*r* = .370, *p* = .009), indicating a more positive interpretation of the clip. In study 2, the average heart rate during the clip was lower than during the preceding (*p* < .05) and following (*p* < .01) non-exposure conditions. Higher trait mindfulness was related to diminished heart rate reactivity (*r* = −.364, *p* = .044) and recovery (*r* = −.415, *p* = .020). This latter effect was obtained only when trait anxiety was used as a statistical covariate. Additionally, increased trait mindfulness was accompanied by higher resting heart rate (*r* = .390, *p* = .027). These outcomes suggest that mindfulness is linked with reductions in negative feelings evoked by violent motion stimuli.

## Introduction

Mindfulness is defined by two components, namely attentional self-regulation and a specific mental approach to personal experience. The self-regulation of attention includes increased focus on personal experience of the present. The mental approach toward personal experience is characterized by acceptance, curiosity, and openness (Bishop et al. 2004). Elevated trait mindfulness has been linked with positive psychological characteristics (Keng et al. 2011), including higher levels of self-esteem (Brown and Ryan 2003), increased positive affect (Garland et al. 2010; Jain et al. 2007; Davidson et al. 2003), and empathy (Lamothe et al. 2016). Practicing mindfulness has similarly been associated with reduced negative psychological characteristics such as anxiety, depression (Goyal et al. 2014), neuroticism (Giluk 2009), and difficulties in emotion regulation (Baer et al. 2006). Furthermore, mindfulness-based techniques are integrated in various psychological therapies, including cognitive behaviour therapy (Singh et al. 2008), dialectical behaviour therapy (Robins and Chapman 2004), and mindfulness-based stress reduction (Kabat-Zinn 2003). A variety of studies has explored the theoretical mechanisms by which mindfulness exerts its positive effects.

Brain imaging studies have suggested that mindfulness is related to changes in neural activity in specific brain circuits. However, the way in which mindfulness impacts upon brain function is influenced by the practitioner’s experience with meditation. In a review of studies, Tang et al. (2015) discussed that novice meditators showed increased task-related activation of the dorsomedial, dorsolateral, ventrolateral, and orbitofrontal prefrontal cortical (PFC) areas and increased resting activity in the anterior cingulate cortex (ACC) and the posterior cingulate cortex (PCC). Conversely, experienced meditators, compared to controls, showed less activation in the PCC and the ventrolateral, dorsolateral, and medial PFC (Lutz et al. 2016b; Tang et al. 2015). However, the activity of the ACC is increased both in experts as it is in novice meditators (Tang and Leve 2016; Tang et al. 2015). The putative explanation for these different patterns of activity is that novice meditators actively recruit “top-down” cognitive processes associated with increased mental effort. Conversely, experts rely more on “bottom-up” processes, linked with the automatization of responses (Chiesa et al. 2013).

Some of the regulatory processes of attention and emotion are mediated in part by brain structures which are affected by mindfulness training. For example, heightened ACC activation can inhibit amygdala responses which can be linked to negative emotionality (Etkin et al. 2006; Petersen and Posner 2012; Wadlinger and Isaacowitz 2011; Ochsner and Gross 2005; Bush et al. 2000). Furthermore, decreased activation in the medial PFC and the PCC was related to diminished self-referential processing, which supports self-detachment, rather than affective or subjective interpretation of personal experience (Lutz et al. 2016a; Brewer et al. 2011; Hölzel et al. 2011; Farb et al. 2007; Gusnard et al. 2001).

Consistent with the findings of brain imaging studies, Tang and Leve (2016) suggested that mindfulness is associated with improved regulatory mechanisms of attention and emotion. All types of emotions can be rated on a Likert scale that ranges in *valence*, from more negative emotional states to more positive emotional states. Irrespective of valence, the intensity of an emotion is referred to as *arousal* (Lang 1995). Various studies indicate that mindfulness differentially affects these two aspects of emotional judgements. Thus, mindfulness increases scores for valence toward more positive emotional states (Remmers et al. 2016; Cameron and Fredrickson 2015; Ho et al. 2015; Taylor et al. 2011; Goldin and Gross 2010) but attenuates physiological arousal, irrespective of whether an emotion is negative or positive (Lin et al. 2016; Brown et al. 2012).

Various studies (Bradley et al. 2012; Bradley 2009; Stanger et al. 2012; Azevedo et al. 2005; Lang and Bradley 2010; Palomba et al. 1997) have demonstrated that the heart rate initially decelerates prominently in response to emotionally valenced static images. Irrespective of the duration of static images, the heart rate returns to pre-exposure values after several seconds. The initial cardiac deceleration can be interpreted as an attentional startle response. The deceleration is greater when negative images are viewed, compared with pleasant and neutral images (Azevedo et al. 2005; Bradley 2009; Palomba et al. 1997). Therefore, cardiac deceleration can be seen as representing a physiological measure of emotional valence (Lang 1995; Palomba et al. 1997). According to Lang and Bradley (2010), and Palomba et al. (1997), the heart rate deceleration in response to negative pictures (e.g., mutilation images) represents an adaptive mechanism, as such stimuli may be related to potential threats to the organism, and is consequently important for survival. Moreover, Bradley (2009) emphasized that less threatening scenes do not require excessive attentional processing, which consequently results in milder cardiac deceleration.

Video clips may capture and sustain attention better than static photographs. Furthermore, the multiple scenes shown during a video clip possibly evoke multiple attentional startle responses. Consequently, the heart rate obtained during the motion clip is expected to be lower than before the clip (Codispoti et al. 2008; Simons et al. 1999). The rate of the heart is largely dictated by the balance of activity of the two branches of the autonomic nervous system (ANS). The parasympathetic, vagally mediated division slows the heart while the sympathetic division speeds it up (Berntson 1997; Levy 1990; Task Force of the European Society of Cardiology 1996). Heart rate variability (HRV), an index of vagal activity, is typically reciprocally related to heart rate (Berntson 1997; Levy 1990; Task Force of the European Society of Cardiology 1996). Hence, when motion images are viewed, an increase in HRV and a decrease in sympathetic activity can be anticipated. Video clips have previously been used to successfully evoke specific emotions, including amusement, disgust, and sadness (Philippot 1993; Gross and Levenson 1995). However, the way in which valence and arousal interact with cardiac responses to video clips is still a matter of debate (Hagenaars et al. 2014; Carvalho et al. 2012).

When watching a positively valenced video, the body tends to move. However, reductions in body movements are observed if the movie is negative or neutral. A positive correlation between reductions in body movements and heart rate was observed for negative video viewing (Hagenaars et al. 2014). This may reflect evolutionary adaptive responses to threatening stimuli whereby reducing body movements can minimize detection by predators (Eilam 2005). These findings taken collectively suggest that the shift in heart rate can be utilized as an index of stimulus aversiveness.

The typical response to distressing images is bi-phasic with the heart initially decelerating and then in a few seconds returning to the pre-exposure level (Lang and Bradley 2010). The cardiac deceleration, in addition to being affected by the valence and arousal of the stimulus, is related to the proximity of the stimulus and some personality traits. Thus, the deceleration is generally evident when the threat is distant. As the danger reaches a threshold of imminent confrontation, or the person exposed to the stimulus has a high level of anxiety, or a specific phobia, a subsequent rise in cardiac activity is observed (Pittig et al. 2013; Thayer et al. 2000; Palomba et al. 2000; Fredrikson 1981). However, the relationship of mindfulness with changes in cardiovascular activity during exposure to aversive stimuli remains poorly understood. In the current study, cardiovascular responses during exposure to a distressing video clip were recorded and analyzed. Here, the movie stimulus did not pose a direct threat and consequently, it was predicted that the average heart rate would be lower during exposure compared to pre-exposure. It was predicted that the overall slowing effect on the heart rate averaged across the 5-min presentation period would be affected by levels of trait mindfulness. However, highly anxious individuals may exhibit a distress response whereby the average heart rate is higher during exposure, compared to before.

The current paper reports two studies. The first study investigated ratings of self-report emotions, valence, and arousal elicited during a 5-min-long violent video clip. The obtained scores for valence and arousal were then related to trait mindfulness. It was hypothesized that mindfulness would be associated with higher responses for valence, but lower arousal ratings. The second study examined changes in the heart rate in relation to the violent video clip and how trait mindfulness is linked to those changes. Contrasting the heart rate obtained before exposure from during exposure to a stimulus provides a measure of cardiac responding. Conversely, contrasting the exposure heart rate with the rate post-exposure gives a measure of cardiac recovery. The cardiac response to negative stimuli is in part influenced by anxiety levels of the participant (Pittig et al. 2013; Thayer et al. 2000). Therefore, trait anxiety was used as a statistical covariate in analyses concerning changes in cardiovascular activity. As noted in the previous paragraphs, mindfulness increases scores for valence and attenuates arousal, whereas heart rate deceleration, typically mediated by increased activity of the vagus, can be used as an index of stimulus aversiveness. Therefore, it was hypothesized that there would be a negative relationship between mindfulness and both heart rate responding to, and recovery from, the emotional stimuli.

Two separate studies were conducted. The first was a group study which examined self-report emotions, valence, and arousal evoked during the projection of a 5-min-long violent video clip. Trait mindfulness scores were obtained before exposure and later related to the measures of valence and arousal. The second study was a laboratory-based exploration of the relationships between cardiovascular responses to the violent video clip and trait mindfulness and anxiety. Informed consent was obtained from all individual participants included in the two studies. Participants received either course credit or a monetary reward of £10 for their participation.

## Study 1

### Method

#### Participants

An undergraduate student sample of 49 participants (28 female and 21 male) aged from 18 to 21 (mean age = 18.86 and *SD* = .84) was recruited from a UK-based university via the local research participation scheme in return for course credit.

#### Procedure

The sample was split in two and tested on two separate occasions. The study took place in a large teaching room with the participants sitting among each other. Upon arrival, everyone was asked to read and sign a consent form, provide demographic information, and provide responses to the Mindfulness Attention Awareness Scale (MAAS) questions. The demographic information included the participants’ age and nationality. Afterwards, the 5-min-long violent video clip was projected on a large screen. After the clip ended, participants provided responses on how they felt during the movie. Debrief was provided by the experimenter at the end of the testing procedure.

#### Measures

Measures used in this study included the MAAS (Brown and Ryan 2003), the self-report emotion questionnaire (Gross and Levenson 1995), a self-report scale used to measure emotions elicited by video content, and measures of valence and arousal as instructed by Lang (1995). Emotions were elicited by a 5-min-long clip containing scenes of graphic violence obtained from popular Hollywood films.

##### Mindfulness Attention Awareness Scale

The MAAS is a self-report, single-factor questionnaire. It measures the presence or absence of concentration on the present internal and external experiences that the authors (Brown and Ryan 2003) believe to be the true essence of trait mindfulness. An example of one of the 15 questions asked is “I rush through activities without being really attentive to them”. Internal validity of this scale in an American psychology student sample (*N* = 313) was *α* = 0.84. In the current study, validity was *α* = 0.79.

##### Self-Report Emotion Questionnaire

The self-report emotion questionnaire (Gross and Levenson 1995) measures 16 positive and negative emotions elicited in response to any video content. Respondents indicate the strength of each emotion on a Likert scale from one to eight. This scale was previously used in a large study assessing over 250 films (Gross and Levenson 1995).

##### Valence and Arousal

Lang (1995) proposed that all emotions can be explained on a two-dimensional spectrum of valence and arousal. In keeping with this view, valence is measured on a scale from − 10 (negative) to + 10 (positive) and arousal is measured from 0 (not aroused) to + 10 (highly aroused). This model was validated by a large emotional picture library and has proven to be reliable.

##### Violent Video Clip

The video clip consisted of scenes from popular Hollywood movies. Scenes included fatal bows to the head, killing by gunshots, armed robbery, mutilation by the use of physical force, and racially aggravated crimes. The content is rated as not to be watched by under 18s.

#### Data Analyses

Descriptive statistics of scores for each emotion were visually examined to determine the type of emotional reaction evoked by the clip. The relationships between trait mindfulness, and self-report scores for valence, and arousal evoked by the clip were correlated with the use of the Pearson’s *r* statistic. This analysis served the understanding of how trait mindfulness influenced the interpretation of the clip content during viewing.

## Results

Ratings of emotions experienced during the video were highest for tension and disgust. Table 1 shows the means, standard deviations, and medians for results of all measures used in study 1. Ratings for valence were mid-range negative, whereas arousal ratings were in the lower part of the scale. The mean score for the MAAS was roughly the same as previously obtained from an undergraduate sample (Brown and Ryan 2003).

**Table 1:** Descriptive statistics for variables used in study 1

	Mean	*SD*	Med
Emotion scale			
Amusement	1.8	1.1	1
Anger	3.22	2.03	3
Arousal	2.1	1.65	1
Confusion	2.67	1.82	2
Contempt	2.2	1.78	1
Contentment	1.37	0.76	1
Disgust	5.1	2.02	5
Embarrassment	1.59	1.14	1
Fear	2.92	2.09	2
Happiness	1.14	0.41	1
Interest	3.27	1.89	3
Pain	3.24	2.17	3
Relief	1.16	0.43	1
Sadness	3.84	2.17	3
Surprise	3.16	2.02	3
Tension	5.14	1.87	5
Valence	− 3.98	3.71	− 5
Arousal	2.71	2.75	2
MAAS	3.67	0.64	3.6

*SD* standard deviation, *Med* median

Trait mindfulness was positively correlated with valence (*r* = .370, *p* = .009) but not with arousal levels (*r* = −.070, *p* = .634) experienced during the 5-min-long clip.

## Study 2

### Method

#### Participants

The study was run using physically active individuals because of their propensity toward good cardiovascular health. A sample of ten females and 23 males were recruited by the researcher at their training facilities. One female terminated the study amid the distress caused by the clip. The final sample consisted of 32 participants, aged between 18 and 31 (mean age = 20.69 and *SD* = 2.80). Inclusion criteria were participation in organized physical activity classes at least four times a week. At the time of the study, 30 participants reported being students and two were in full-time employment. Individuals were rewarded with a payment of £10.

#### Procedure

This study had two parts. In the first part, participants were asked to fill out the self-report questionnaires. The second part entailed collecting cardiovascular data immediately before, during, and immediately after a 5-min-long violent video clip.

Physically active individuals were approached at their training facilities, and the purpose of the study was explained to them. Those who were willing to participate (*N* = 32) gave informed consent the following week just prior to completing the personality inventories. After completion, session arrangements were made for the participants to attend the second, laboratory-based part of the study.

The cardiovascular parameters were assessed using the Biocom 4000 electrocardiogram (ECG). The assessment took place in a laboratory, where the lights were dimmed and no significant distractors were present. On the day of testing, participants were asked to refrain from both exercise and potential stimulants. Participants were asked to sit quietly for 5 min before cardiovascular assessment commenced which allowed the individual to acclimatize to the room environment. Next, the participants were instructed on the testing procedure. During the explanation, a silver/silver chloride electrode was placed on each participant’s wrist, elastic sweat bands were used to prevent movement.

The ECG trace was collected over three 5-min-long time intervals: immediately before, during, and immediately after the violent video clip. Before exposure to the video clip, the participants sat quietly while their cardiac activity was measured for 5 min. After the 5 min of testing, the video clip was displayed on a laptop monitor and another 5-min testing interval started. For a more detailed description of the content of the clip, please refer to study 1 “Method” section. The final 5 min of cardiovascular activity was obtained immediately after the clip ended. This collectively gave a continuous 15 min of ECG recording. At the end of the testing procedure, the participant was given feedback on the study and some basic information about the ECG results.

Recorded traces were scanned for artefacts using the Biocom Heart Rate Scanner Professional Edition software installed on a Samsung laptop. The quality of each trace was visually checked for artefacts. Where possible, artefacts were identified and replaced with interpolated R-R intervals with the use of an automated software-based algorithm. A trace was rejected if a signal noise prevented accurate visual localization of the “R” wave, artefacts were too difficult to correct, or when the software deemed the recording to be poor quality. For each 5-min-long ECG trace, the average heart rate and root mean square of successive heartbeat interval differences (rMSSD), a reliable measure of HRV (Task Force of the European Society of Cardiology 1996), were calculated with the use of the dedicated software.

#### Measures

Mindfulness was measured with the MAAS, already described in study 1. In the current study, Cronbach’s alpha for MAAS was *α* = 0.83. In addition, the trait subscale of the State-Trait Anxiety Inventory (STAI) (Spielberger et al. 1983) was used. The same 5-min video clip described in the previous study was used here.

##### State-Trait Anxiety Inventory

Anxiety was measured with the STAI (Spielberger et al. 1983), a popular anxiety self-report measure. It contains 20 items for the state and 20 items for the trait subscales. For the purposes of assessing anxiety in physically active participants only, the trait anxiety scale was used. Spielberger et al. (1983) report that Cronbach’s alpha for a mixed-gender college student population is *α* = 0.91. Reliability for the current study was also *α* = 0.91.

#### Data Analyses

The main effects of the video manipulation on the heart rate and heart rate variability (rMSSD) were analyzed with the repeated measures ANOVA within the subject’s effects. The differences in cardiovascular activity between the three time intervals were further analyzed with the repeated measures planned contrasts statistic. Therefore, the resting heart rate was compared with the heart rate during exposure. Similarly, the heart rate during exposure was contrasted with the heart rate after exposure. Corresponding contrasts were obtained for rMSSD before, during, and after the violent clip. Effect sizes are expressed as partial eta squared [*η* ^2^_p_] with the following recommended norms for interpretation: small = 0.01, medium = 0.06, and large = 0.14. Next, to examine the effects of mindfulness and trait anxiety, for each participant, the strength of shifts in cardiovascular activity was calculated. This was done by subtracting the values of time interval variables from each other. This resulted in forming of four new variables, two for shifts in heart rate and two for shifts in heart rate variability. Two variables denoting the change from before to during the clip were given an idiom, *reactivity*, whereas the other two, *recovery*. Higher values indicate greater shifts in cardiovascular activity. These new variables, as well as resting heart rate (HR) and HRV, were later correlated with trait mindfulness and anxiety with the Pearson’s *r* statistic. Lastly, partial correlations between trait mindfulness and shifts in cardiovascular activity using trait anxiety as a covariate were performed. The same analysis was performed for trait anxiety and cardiovascular shifts when trait mindfulness was entered as a covariate. The rationale for controlling for these variables as covariates is explained in the “Introduction” section.

## Results

For the current sample of *N* = 32, mean resting HR was comparable to normative values (*M* = 80 bpm). Resting rMSSD (HRV) values were higher than the mixed-gender norms (rMSSD: *M* = 42, *SD* = 15) reported by Nunan et al. (2010). Descriptive statistics for trait mindfulness (*M =* 3.86, *SD* = .69) and anxiety (*M* = 41.94, *SD* = 10.40) were roughly the same as previously published values (Brown and Ryan 2003; Spielberger et al. 1983). Table 2 shows the means and standard deviations for cardiovascular variables before, during, and after exposure to the clip.

**Table 2:** Descriptive statistics for cardiovascular activity before, during, and after the clip

	Before		During		After	
	*M*	*SD*	*M*	*SD*	*M*	*SD*
HR	75.76	12.81	73.87	13.93	76.69	12.41
HRV	48.69	30.47	57.82	46.63	47.10	33.27

*M* mean, *SD* standard deviation

Mauchly’s test indicated that the assumption of sphericity had not been violated (*χ* ^2^ (2) = 0.99, *p* = .87). The effect of video manipulation on mean HR was significant (*F*(2,62) = 6.70, *p* < .01, *η* ^2^_p_ = 0.18). Additional examination of planned contrasts revealed that the average HR obtained during the 5-min motion clip was slower than in the preceding (*F*(1,31) = 5.39, *p* < .05, *η* ^2^_p_ = 0.15) and following (*F*(1,31) = 12.68, *p* < .01, *η* ^2^_p_ = 0.29) non-exposure 5-min-long conditions.

Mauchly’s test indicated that the assumption of sphericity had been violated (*χ* ^2^ (2) = 0.67, *p* = 0.002); therefore, the Greenhouse-Geisser correction is reported. The effect of video manipulation on HRV was significant (*F*(2,62) = 3.79, *p* < .05, *η* ^2^_p_ = 0.11). Further examination of planned contrasts revealed that HRV did not differ before compared to during exposure to the violent video clip (*F*(1,31) = 2.97, *p* = .10 *η* ^2^_p_ = 0.09), but immediately following the clip, HRV significantly decreased (*F*(1,31) = 9.58, *p* < .01, *η* ^2^_p_ = 0.24).

Trait mindfulness had a positive correlation with resting HR and a negative correlation with HR recovery. The later outcome suggests a relationship between high mindfulness and diminished shift in average HR between during and after the video clip. As expected, trait anxiety had a negative correlation with heart rate reactivity, which suggests that for highly anxious individuals, the HR deceleration effect is less conspicuous. The remaining correlations were not significant. Table 3 contains the outcomes discussed in this paragraph.

**Table 3:** Pearson’s zero-order correlations between psychological characteristics and cardiovascular activity before exposure and in phases of reactivity and recovery

	HR before	HR reactivity	HR recovery	HRV before	HRV reactivity	HRV recovery
MAAS	0.390* (0.027)	− 0.129 (0.480)	− 0.372* (0.036)	− 0.228 (0.210)	− 0.248 (0.172)	− 0.311 (0.083)
Trait anxiety	− 0.070 (0.703)	− 0.387* (0.029)	0.001 (0.997)	0.120 (0.514)	0.1302 (0.472)	− 0.097 (0.597)

The “*p*” values are in the parentheses

*p < .05

Table 4 contains partial correlations for psychological characteristics and the shifts in cardiovascular activity. The rationale for using trait anxiety as a covariate is described in the “Introduction” section. When trait anxiety was used as a covariate, trait mindfulness had a negative correlation with both variables representing a shift in the average heart rate, that is, HR reactivity and recovery. This suggests that trait mindfulness is associated with an overall diminished slowing effect on HR in response to the clip. Additionally, trait mindfulness and HRV recovery were negatively correlated, meaning that HRV recovery is weaker in participants scoring high on mindfulness. Similarly, when trait mindfulness was entered as a covariate, anxiety had a negative correlation with HR reactivity. The remaining variables were not significantly correlated.

**Table 4:** Partial correlations between personality characteristics and shifts in cardiovascular activity

	HR reactivity	HR recovery	HRV reactivity	HRV recovery
MAAS	− 0.364* (0.044)	− 0.415* (0.020)	− 0.213 (0.250)	− 0.397* (0.027)
Trait anxiety	− 0.500** (0.004)	− 0.197 (0.287)	0.025 (0.892)	− 0.276 (0.133)

Anxiety was entered as a covariate out when mindfulness was correlated and vice versa; the “*p*” values are in the parentheses

*p < .05; **p < .01

## Discussion

Mindfulness can be defined as a process of regulating attention in a way that promotes acceptance of present moment personal experience (Bishop et al. 2004). This definition has driven us to hypothesize that increased trait mindfulness relates to a less negative interpretation of aversive stimuli. The results of study 1 showed that participants rated a violent video clip as mildly arousing and aversive, whereas ratings of specific emotions were highest for tension and disgust. Individuals scoring higher on trait mindfulness rated the clip as less aversive. Moreover, study 2 showed that the average HR was lower during the clip and returned to pre-exposure values immediately after exposure. This is in accordance with the HR deceleration observed in response to aversive static (Lang et al. 1993) and motion images (Palomba et al. 2000) that evoked feelings of disgust. The change in vagally mediated HRV did not reach a statistically significant level. This suggests that the HR deceleration was not specific to the vagal influences on HR and might have been mediated by sympathetic activity, or resulted from an interplay of the two systems. The HR deceleration was weaker for participants scoring higher on trait mindfulness. In addition to the hypothesized results, we found a positive correlation of trait mindfulness with resting HR. We conclude that increased mindfulness is associated with a less negative interpretation of aversive stimuli.

Theories of mindfulness have emphasized the regulatory processes that guide attention. More specifically, these processes may entail sustained concentration, fluent shifting of focus, and inhibition of excessive elaboration of thoughts, emotions, and sensations (Bishop et al. 2004). Typically, when attention is directed toward a mildly aversive emotion evoking stimulus, the HR slows. The deceleration is greater in response to negative, compared with pleasant and neutral stimuli. In the current study, when trait anxiety scores were used as a statistical covariate, trait mindfulness had an action on attention-induced changes in HR. This relationship between mindfulness and attention was expressed in a dampening of the commonly observed slowing of the heart in response to viewing of a negative stimulus. Our results suggest that mindful attention dampens the typically observed cardiac response. This pattern of results therefore suggests that the mindful participant is attending with focus but interprets the stimulus as less negative.

An alternative interpretation of these results is that mindfulness is associated with reduced sensory intake during viewing of aversive motion images. The concomitants of orientating associated cardiovascular changes remain poorly understood. Bradley (2009) critically analyzed a series of studies on HR deceleration in response to static emotion eliciting stimuli. She described the phenomenon as cardiac orienting related to sensory intake, whereby a more pronounced deceleration indexes enhanced perceptual focus. The understanding that cardiac orienting is related to increased sensory intake is supported by studies exploring memorization of emotional stimuli. These studies show that greater cardiac deceleration is associated with improved recall for emotional pictures and words (Abercrombie et al. 2008; Buchanan et al. 2006). Conversely, mindfulness was previously found to attenuate recall for negative words (Alberts and Thewissen 2011). In our study, higher scores on trait mindfulness are related to weaker cardiac slowing when watching the violent video clip. This suggests that mindfulness is related to a decrease in sensory intake of stimuli evoking negative feelings. However, whether these propensities hold true for video clips requires further experimental assessment.

Although the interpretation that mindfulness is associated with reduced sensory intake may appear to be at odds with descriptions of mindfulness that emphasize attention and awareness in the present moment, it should be noted that the mindfulness measure used in this study, the MAAS, assesses mindfulness along a single dimension. As such, it is possible that while some components of mindfulness may be associated with reduced sensory intake, other components, e.g., the putative *Acceptance* scale of the Freiburg Mindfulness Inventory (FMI) (Walach et al. 2006; Sauer et al. 2011a), may be associated with the converse pattern. Indeed, previous studies using the FMI measure that assesses various components of mindfulness found differing relations of the *Presence* subscale and the Acceptance subscale with anxiety, depression, valence of words, and Stroop reaction times, a test of higher-order brain function associated with activity of the ACC (Kohls et al. 2009; Sauer et al. 2011b). The potentially contrasting relations of different components of mindfulness with cardiac deceleration and reduced sensory intake are worthy of future investigation.

Shifts in HR in response to environmental cues may serve an evolutionary advantage of freezing when challenged. This response can be characterized by heightened stimuli intake, reductions in body movements, and other physiological changes (Korte et al. 2005). These physiological changes purportedly increase the chance of survival when danger occurs. Together with the slowing of the heart, freezing was observed when individuals were exposed to distressing static and motion images (Azevedo et al. 2005; Facchinetti et al. 2006; Vila et al. 2007). These studies support the notion that mindfulness is related to a less negative interpretation of aversive stimuli.

Activity in brain areas, such as the mPFC, ACC, and insula, has been shown to be related to both mindfulness and attention (Petersen and Posner 2012; Tang et al. 2015). The ACC has been shown to have a high level of resting state activity in both novice and expert meditators (Tang et al. 2015). It has been observed by Etkin et al. (2006) that during a task that involved resolution of emotional conflict, the rostral ACC (rACC) activity was inversely related to an activity in the right amygdala. Both the strength of this inverse relationship and the dampened autonomic responsivity were related to increased performance on the emotional conflict resolution task. Furthermore, another study argued that the grey matter volume of the right amygdala is decreased in individuals with high trait mindfulness (Taren et al. 2013). Hence, the underlying brain structure and function seen in mindful individuals may also lend support to the conclusion that mindfulness is associated with a less negative interpretation of emotional stimuli.

Surprisingly, we have found that like more mindful individuals, the more anxious participants also show similar patterns of HR reactivity when one would expect these constructs to diverge. The similarity in the direction of these relationships is likely driven by different underlying mechanisms. Anxiety might have a positive relationship to affective reactivity, while the converse link was previously observed between mindfulness and affective reactivity (Ostafin et al. 2014). Furthermore, the different patterns might be masked by the correlational procedures applied in the current study. The distinct effects of mindfulness and trait anxiety on cardiac responses to aversive stimuli represent an area worthy of future investigation.

We have shown that the positive effects of mindfulness are associated with higher levels of resting HR. This result has been previously found in other types of meditative practices (Peng et al. 1999, 2004). This is paradoxical in that high resting HR is normally associated with increased all-cause mortality and various coronary illnesses (Fox et al. 2007; Ho et al. 2014). Conversely, although low resting HR is associated with good physical health, it is also strongly linked to many types of offending, including violent and non-violent crimes but not sex offences (Latvala et al. 2015; Ortiz and Raine 2004; Portnoy and Farrington 2015). Increased mindfulness has previously been associated with reduced aggressiveness (Borders et al. 2010; Heppner et al. 2008; Singh et al. 2003) and has been suggested as a potential therapeutic intervention for improving emotion regulation among sexual offenders (Gillespie et al. 2012). The current pattern of results might suggest that mindfulness may be linked to reductions in aggressive behaviour via intrinsic, physiological mechanisms. However, the mediating effects of cardiovascular parameters on the inverse relationship of mindfulness with aggression are beyond the scope of the current paper and may represent a potentially interesting avenue for future research.

### Limitations

The participants were not asked about their actual experience with mindfulness techniques. This leaves open the possibility that the trait measured with the MAAS does not resemble actual mindfulness experience but only a unique quality of consciousness possessed by the participants (Brown and Ryan 2003). The findings are also based on a relatively small sample, and this may have implications for statistical power and effect size estimates (Button et al. 2013).; Kühberger et al. 2014). As such, while the current results represent an interesting advance on the understanding of the relationship between trait mindfulness and physiological mechanisms, they should be confirmed in a larger sample. Finally, the cardiovascular measures were obtained from a sample of physically active individuals. Cardiac activity and patterns of responding and recovery may differ in individuals who do not engage in physical exercise.

The current studies evaluated the relationship between trait mindfulness and emotional and cardiac responding to a non-imminent threat, that is, an aversive, violent video clip. Outcomes indicate that individuals scoring high on trait mindfulness felt less negative emotions while viewing the clip and exhibited diminished cardiovascular slowing in response to the clip. Higher trait mindfulness was also associated with increased resting HR. Altogether, these results suggest that mindfulness is related to reductions in negative feelings while attending to a violent motion stimulus. Speculative mechanisms upon which mindfulness acts to reduce negative emotionality are the decreased intake of negative emotional stimuli, demonstrated here by diminished cardiac orienting.
